# In-Depth Characterization of the *Staphylococcus aureus* Phosphoproteome Reveals New Targets of Stk1

**DOI:** 10.1074/mcp.RA120.002232

**Published:** 2021-01-11

**Authors:** Nadine Prust, Saar van der Laarse, Henk W.P. van den Toorn, Nina M. van Sorge, Simone Lemeer

**Affiliations:** 1 Biomolecular Mass Spectrometry and Proteomics, Bijvoet Center for Biomolecular Research and Utrecht Institute for Pharmaceutical Sciences, Utrecht University, Utrecht, the Netherlands; 2 Netherlands Proteomics Center, Utrecht, the Netherlands; 3 Medical Microbiology, University Medical Center Utrecht, Utrecht University, Utrecht, the Netherlands; 4 Department of Medical Microbiology and Infection Prevention and Netherlands Reference Laboratory for Bacterial Meningitis, Amsterdam University Medical Center, University of Amsterdam, Amsterdam, the Netherlands

**Keywords:** Microbiology, phosphorylation, bacteria, pathogens, mass spectrometry, serine/threonine kinases, phosphatases, substrate identification, CAA, 2-chloroacetamide, eSTK, eukaryotic-type serine/threonine kinase, FDR, false discovery rate, HCD, higher-energy collision-induced dissociation, IMAC, immobilized metal ion affinity chromatography, LC, liquid chromatography, MOAC, metal oxide affinity chromatography, MRSA, methicillin-resistant *Staphylococcus aureus*, PTM, posttranslational modification, RR, response regulator, SDC, sodium deoxycholate, TCEP, tris(2-carboxyethyl)phosphine, TCS, two-component system

## Abstract

*Staphylococcus aureus* is a major cause of infections worldwide, and infection results in a variety of diseases. As of no surprise, protein phosphorylation is an important game player in signaling cascades and has been shown to be involved in *S. aureus* virulence. Albeit long neglected, eukaryotic-type serine/threonine kinases in *S. aureus* have been implicated in this complex signaling cascades. Due to the substoichiometric nature of protein phosphorylation and a lack of suitable analysis tools, the knowledge of these cascades is, however, to date, still limited. Here, were apply an optimized protocol for efficient phosphopeptide enrichment *via* Fe^3+^-IMAC followed by LC-MS/MS to get a better understanding of the impact of protein phosphorylation on the complex signaling networks involved in pathogenicity. By profiling a serine/threonine kinase and phosphatase mutant from a methicillin-resistant *S. aureus* mutant library, we generated the most comprehensive phosphoproteome data set of *S. aureus* to date, aiding a better understanding of signaling in bacteria. With the identification of 3800 class I p-sites, we were able to increase the number of identifications by more than 21 times compared with recent literature. In addition, we were able to identify 74 downstream targets of the only reported eukaryotic-type Ser/Thr kinase of the *S. aureus* strain USA300, Stk1. This work allowed an extensive analysis of the bacterial phosphoproteome and indicates that Ser/Thr kinase signaling is far more abundant than previously anticipated in *S. aureus*.

## Highlights

• An optimized phosphopeptide enrichment protocol for Gram-positive bacteria
• Extensive protein phosphorylation in *Staphylococcus aureus*
• Seventy-four downstream targets of the kinase Stk1 in *S. aureus* were identified
• Extensive phosphorylation indicates existence of other kinases transferases

## Graphical Abstract

**Figure:**
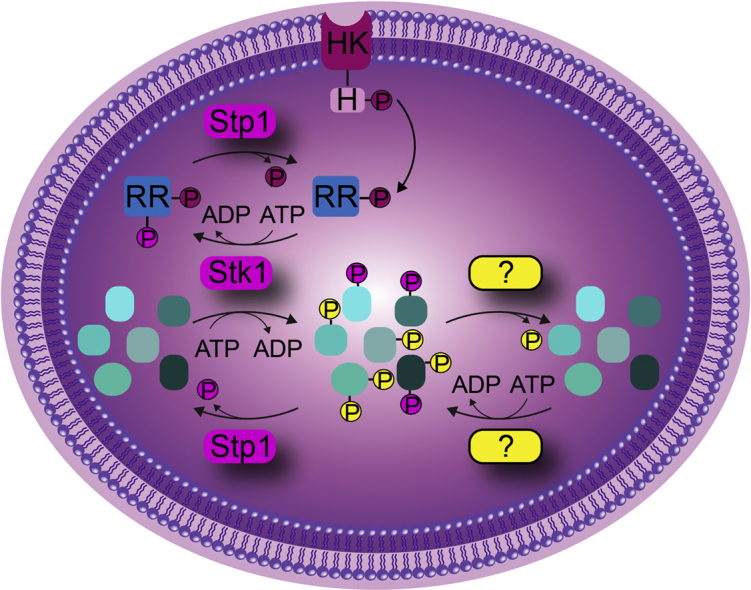

## In Brief

An optimized workflow for phosphopeptide enrichment of Gram-positive bacteria has been developed, revealing extensive protein phosphorylation in *S. aureus*. Using mutant strains and quantitative proteomics, 74 downstream targets of the kinase Stk1 were identified in *S. aureus*. Given the total number of 3800 identified phosphosites, other, yet unannotated, kinases are expected to exist in *S. aureus*.

Protein phosphorylation is a major regulator of cellular processes in all kinds of organisms. Eukaryotic protein phosphorylation commonly occurs on serine (S), threonine (T), and tyrosine (Y) residues, and it was long assumed that similar posttranslational modifications (PTMs) only play a minor role in prokaryotes. Instead, it was proposed that protein histidine (H) phosphorylation, as, for example, used in the so-called two-component systems (TCSs), is the main regulatory PTM (1, 2). The discovery of eukaryotic-type serine/threonine kinases (eSTKs) and the identification of numerous pS/pT peptides changed this perception. The occurrence of STY phosphorylation has now been identified in both Gram-negative and Gram-positive bacteria including important human pathogens (2, 3).

The substoichiometric nature of protein phosphorylation not only necessitates a highly efficient sample preparation but also a highly efficient enrichment method before mass spectrometric analysis. To date, a lack of efficient methods to enrich for the substoichiometric phosphopeptides from Gram-positive bacteria confined the information about signaling pathways in these bacteria. Most enrichment techniques exploit the affinity of negatively charged phosphopeptides toward metal oxides such as TiO_2_, which is mostly used for metal oxide affinity chromatography (MOAC) (4, 5), or metal ions such as Ti^4+^ and Fe^3+^ that are used for immobilized metal affinity chromatography (IMAC) (6, 7). Recent developments in enrichment strategies as well as fragmentation, mass spectrometric detection, and phosphorylation site localization assessment allow the identification of thousands of phosphorylation sites (8, 9, 10). However, we recently showed that contamination by DNA/RNA and phospholipids in standard sample preparation protocols hampers the Fe^3+^-IMAC enrichment (11). Consequently, removing those contaminants drastically improved the identification for the Gram-negative bacterium *Escherichia coli* (11). This improvement has encouraged us to also further optimize the sample preparation for Gram-positive bacteria, which require a more stringent cell lysis due to the presence of the thick peptidoglycan layer.

This optimization of phosphopeptide identification from Gram-positive bacteria could subsequently help to understand signaling pathways of important human pathogens such as *Staphylococcus aureus* (*S. aureus*). *S. aureus* is a great threat to public health since it is a major cause of infections worldwide (12, 13, 14). Due to a variety of virulence factors and its exceptional versatility, this pathogen is able to cause a broad spectrum of infections ranging from mild skin infections to life-threatening infections such as toxic shock syndrome and sepsis (13). From a clinical point of view, the evolution of antibiotic resistant strains (*e.g.*, methicillin-resistant *S. aureus* (MRSA)) is complicating both prevention and treatment of *S. aureus* infections, rendering *S. aureus* a high-priority pathogen according to the World Health Organization (15). In the search for new treatment strategies, major efforts have been devoted to elucidating the virulence and adaptation mechanisms of this pathogen (16, 17, 18). Nevertheless, we are still far from a comprehensive understanding of the underlying mechanisms involved in the intracellular signaling pathways engaged in virulence.

*S. aureus* contains a large variety of virulence factors, which allows bacterial adaptation and survival in a temporal (19) and host tissue-specific manner (20). Gene expression is regulated through sophisticated regulatory mechanisms, including protein phosphorylation *via* TCSs (16). TCSs sense specific environmental changes such as pH or nutrient concentrations and translate these extracellular stimuli into intracellular responses by affecting gene transcription, amongst which are virulence factors (21). *S. aureus* encompasses 16 to 18 known TCSs that are known to be involved in virulence gene regulations, cell wall metabolism, nutrient sensing, and response to antimicrobial agents (22, 23). In addition to these TCSs, *S. aureus* USA300 also expresses two serine kinases (HprK and RsbW) and one eSTK (24, 25, 26), most importantly serine/threonine kinase 1 (Stk1, also known as PknB, protein kinase B). Stk1 and its corresponding phosphatase, Stp1, have been shown to fine-tune the response of certain TCS response regulators (RR) by adding/removing additional phosphate on serine/threonine residues (22). Such regulation is clinically relevant since phosphorylation of the RRs VraR and GraR by Stk1 has been linked to vancomycin resistance (24). Here, we optimized the sample preparation workflow for phosphoproteomic studies of Gram-positive bacteria. This optimized workflow was first tested on the nonpathogenic model organism *Bacillus subtilis* and subsequently applied to *S. aureus*. Moreover, we performed a label-free quantitative (LFQ) phosphoproteomics study and identified 74 potential new targets of Stk1 and Stp1, which will help in elucidating the role Stk1 and Stp1 in signal transduction and virulence. Furthermore, our data shows that more, unknown, Ser/Thr kinases, are involved in signaling in *S. aureus.*

## Experimental Procedures

### Bacterial Culture

*B. subtilis* 168 was grown overnight in 50 ml Luria broth (LB) at 37 °C with agitation in n = 3 biological replicates. Transposon mutants NE98 (disruption in unrelated surface protein encoding gene *sdrE*), NE217(disruption in protein coding gene *pknB*), and NE1919 (disruption in gene SAUSA300_1112 encoding Stp1), all containing an erythromycin resistance marker, of the *S. aureus* USA300 JE2 strain were obtained from the Nebraska Transposon Mutant Library (NTML) (27). Mutants were grown in n = 4 biological replicates, overnight in 25 ml Todd Hewitt broth (THB) supplemented with 5 ug/ml erythromycin at 37 °C with agitation. Bacteria were harvested by centrifugation (15 min, 3200 rpm at 4 °C), and the supernatant was subsequently removed.

### Optimized Cell Lysis

Bacterial cell lysis was performed as described in Potel *et al.* (2018) (11) with optimization for Gram-positive bacteria. One volume bacteria pellet was resuspended in five volumes of lysis buffer (100 mM Tris-HCl pH 8.5, 7 M Urea, 5 mM tris(2-carboxyethyl)phosphine (TCEP), 30 mM 2-Chloroacetamide (CAA), 10 U/ml DNase I, 1 mM magnesium chloride (Sigma-Aldrich, Steinheim, Germany), 1% (v/v) benzonase (Merck Millipore, Darmstadt, Germany), 1 mM sodium orthovanadate, phosphoSTOP phosphatases inhibitors (Roche), and complete mini EDTA-free protease inhibitors). The lysis was performed by bead beating for 17.5 min (1.5 min on, 2 min off) at 2850 rpm (Disruptor Genie, Scientific industries) in case of *B. subtilis* and 3200 rpm (Mini-Beadbeater-24, Bio Spec Products Inc) for *S. aureus*. Subsequently, the beads were pelleted by centrifugation (2 min at 3000 rpm) and 1% (v/v) Triton X-100 in case of *B. subtilis* and 1% (v/v) Triton X-100 plus 1% (v/v) sodium deoxycholate (SDC, final concentration) in case of *S. aureus* were added to the bacteria lysate. Complete lysis was reached by sonication for 45 min (20 s ON, 40 s off) using a Bioruptor Plus. Cell debris was removed by ultracentrifugation (45,000 rpm for 1 h at 4 °C). Protein concentration of the supernatant was determined *via* a Bicinchonic Acid (BCA) assay. To decrease the SDC concentration to <0.4%, the supernatant was diluted 2.5 times with dilution buffer (100 mM Tris-HCl pH 8.5, 7 M Urea, 5 mM TCEP, 30 mM CAA, 1 mM magnesium chloride (Sigma-Aldrich, Steinheim, Germany), 1 mM sodium orthovanadate, phosphoSTOP phosphatases inhibitors (Roche), and complete mini EDTA-free protease inhibitors). One percent (v/v) benzonase was added to the supernatant mixture and incubated for 2 h at room temperature. Subsequently, methanol/chloroform precipitation was performed as described earlier (11). The precipitate was then resuspended in digestion buffer (100 mM Tris-HCL pH 8.5, 30 M CAA, 1% (v/v) SDC (Sigma-Aldrich), and 5 mM TCEP). Protein digestion was performed overnight at room temperature using a mix of trypsin and Lys-C in a ratio of 1:25 and 1:100 (w/w), respectively. Protein digests were acidified to pH 3.5 using 10% formic acid (Sigma-Aldrich), and precipitated SDC was removed by centrifugation (1400 rpm, 5 min). The supernatant was loaded onto C18 Sep-Pak (3 cc) resin columns (Waters) for desalting. The loaded samples were washed twice with 0.1% (v/v) formic acid, and bound peptides were eluted with 600 μl 40% acetonitrile and 0.06% formic acid. Eluted peptides were split into 2 mg fractions, and samples for full proteome analysis were frozen in liquid nitrogen and freeze-dried.

### Phosphopeptide Enrichment

Fe^3+^-IMAC enrichments were performed as previously described (11). In short, 2 mg lyophilized peptides were resuspended in loading buffer A (30% acetonitrile and 0.07% TFA) and, if necessary, the pH was adjusted to 2.3 using 10% TFA. The samples were loaded onto the Fe^3+^-IMAC column (Propac IMAC-10 4 × 5 mm column, Thermo Fischer Scientific). Bound phosphopeptides were eluted with elution buffer B (0.3% NH_4_OH). The respective gradient is described in supplemental Table S1. The UV-abs signal at a wavelength of 280 nm was recoded at the outlet of the column and eluting phosphopeptides were collected manually. Subsequently, phosphopeptides were frozen in liquid nitrogen and freeze-dried.

### LC-MS/MS

Nanoflow LC-MS/MS analysis was performed using an Agilent 1290 (Agilent technologies, Middelburg, The Netherlands) coupled to an Orbitrap Q-Exactive HF-X (Thermo Fisher Scientific, Bremen, Germany). Lyophilized phosphopeptides or full proteome samples were resuspended in 20 mM citric acid (Sigma-Aldrich), 1% (v/v) formic acid, or 2% (v/v) formic acid, respectively. Resuspended phosphopeptides, corresponding to 1.6 mg or 200 ng full proteome samples, were injected, trapped, and washed on a trap-column (100 μm i.d. × 2 cm, packed with 3 μm C18 resin, Reprosil PUR AQ, Dr Maisch, packed in-house) for 5 min at a flow rate of 5 μl/min with 100% buffer A (0.1 FA, in HPLC grade water). Peptides were subsequently transferred onto an analytical column (75 μm × 60 cm Poroshell 120 EC-C18, 2.7 μm, Agilent Technology, packed in-house) and separated at room temperature at a flow rate of 300 nl/min using a 85 min linear gradient from 8% to 32% buffer B (0.1% FA, 80% ACN) or a 115 min linear gradient from 13% to 44% buffer B. Electrospray ionization was performed using 1.9 kV spray voltage and a capillary temperature of 320 °C. The mass spectrometer was operated in data-dependent acquisition mode: full scan MS spectra (m/z 375–1600) were acquired in the Orbitrap at 60,000 resolution for a maximum injection time of 20 ms with an AGC target value of 3e6 charges. Up to 12 precursors for phosphoproteome samples and up to 15 precursors for full proteome samples were selected for subsequent fragmentation. High-resolution HCD MS2 spectra were generated using a normalized collision energy of 27%. The intensity threshold to trigger MS2 spectra was set to 2e5, and the dynamic exclusion to 12 or 16, respectively. MS2 scans were acquired in the Orbitrap mass analyzer at a resolution of 30,000 (isolation window of 1.4 Th) with an AGC target value of 1e5 charges and a maximum ion injection time of 50 ms. Precursor ions with unassigned charge state as well as charge state of 1+ or superior/equal to 6+ were excluded from fragmentation.

### Data Analysis

Raw files were processed using MaxQuant software (version 1.6.3.4), and the Andromeda search engine was used to search against either a *B. subtilis* 168 (Uniprot/TrEMBL, December 2017, 4247 entries) or *S. aureus* USA300 database (Uniprot, June 2018, 5954 entries) with the following parameters for phosphoproteome analysis: trypsin digestion with a maximum of three missed cleavages, carbamidomethylation of cysteines (57.02 Da) as a fixed modification, methionine oxidation (15.99 Da), N-acetylation of proteins N-termini (42.01 Da), and phosphorylation on serine, threonine, tyrosine, and histidine residues (79.96 Da) as variable modifications. Mass tolerance was set to 4.5 ppm at the MS1 level and 20 ppm at the MS2 level. The false discovery rate (FDR) was set to 1% for peptide-spectrum matches (PSMs) and protein identification using a target-decoy approach, a score cutoff of 40 was used in the case of modified peptides, and the minimum peptide length was set to seven residues. The match between run features was enabled with a matching time window of 0.7 min and an alignment time window of 20 min. The MaxQuant generated tables “evidence.txt” and “phospho (HSTY)Sites.txt” were used to calculate the number of unique phosphopeptides and phosphosites identified, respectively, and known contaminants were filtered out. For full proteome analysis, the following deviations were applied: trypsin digestion with a maximum of two missed cleavages, carbamidomethylation of cysteine’s (57.02 Da) as a fixed modification, methionine oxidation (15.99 Da), N-acetylation of protein N-termini (42.01 Da) as variable modifications. Relative label-free quantification was performed using the MaxLFQ algorithm with the minimum ratio count set to 2.

### Identification of DNA/RNA Contamination in MS2 Spectra

Raw files were converted into.mgf with Proteome Discoverer (Vers. 2.3.0.523), using deisotoping with an isotope deviation tolerance of 25 mmu. Subsequently mgf-files were analyzed using an in-house made script searching MS2 spectra for a 330.06 m/z peak with a 0.02 Da tolerance.

### Statistical Data Analysis

For the phosphoproteome and full proteome analysis, the MaxQuant generated “phospho (HSTY)Sites.txt” and “proteinGroups.txt” file, respectively, were used for subsequent statistical data analysis in R studio (R version 3.6.0). Four biological replicates per mutant were analyzed. The data was filtered for “Reversed” and “Potential contaminant.” In case of the phosphoproteome, an Andromeda localization score greater than 0.75 was required. Intensities for the phosphoproteome data, or LFQ intensities in case of the full proteome analysis, were log2 transformed. For each phosphosite or protein, the median calculated per sample was subtracted to compensate for systematic measurement effects. Only proteins with at least three valid values in one condition and two valid values in at least one other condition. Data was checked for normal distribution before one-way ANOVA on each phosphosite or protein was done, after which the *p*-values were adjusted with the Benjamini–Hochberg procedure. The post-hoc Tukey honestly significant difference (HSD) method was used to identify changing p-sites between the individual groups. A Tukey HSD *p*-value cutoff of 0.05 and a fold change cutoff of the mean ± one standard-deviation of the data were used to select for significantly changing phosphosites or proteins between two groups.

### Experiment Design and Statistical Rationale

Each sample was grown in n = 4 biological replicates, enriched and injected separately into the LC-MS/MS system. Each raw file was separately processed using the MaxQuant software. This analysis was sufficient to saturate the number of phosphosites detected.

### Phosphosite Environment Analysis

Phosphosites listed in “phospho (HSTY)Sites.txt” with a localization probability of at least 0.75 were used for further analysis in R. To assess whether phosphorylation events preferentially occurred on flexible regions of proteins, the relative occurrence of amino acids present in a window of five amino acids before and after the phosphosites was computed for *S. aureus*, *E*. *coli* and human phosphoproteomes. The resulting amino acid frequencies were grouped into five groups, namely flexible (A/G/P), acidic (D/E), basic (K/R), aromatic (F/W/Y), and others. To investigate the conservation of *S. aureus* phosphosites across different organisms, the identified phosphoproteins were mapped back to their corresponding gene names (Uniprot retrieve ID/mapping tool). The PSP database (downloaded may 8th, 2020) was also mapped back to gene names and for all gene names found in both the MS data and PSP database, all corresponding proteins in both sets were aligned (msa package available *via* Bioconductor, http://www.bioinf.jku.at/software/msa/ (28)) using the ClustalW algorithm with default settings. For each gene name, identity scores were computed by calculating the fraction of fully conserved amino acids across the whole alignment (*i.e.*, the presence of gaps in one of the aligned protein sequences reduces the identity score). Scripts can be made available upon request.

## Results and Discussion

### Enhanced Phosphopeptide Identification for Gram-Positive Bacteria B. subtilis and S. aureus

**Fig. 1:**
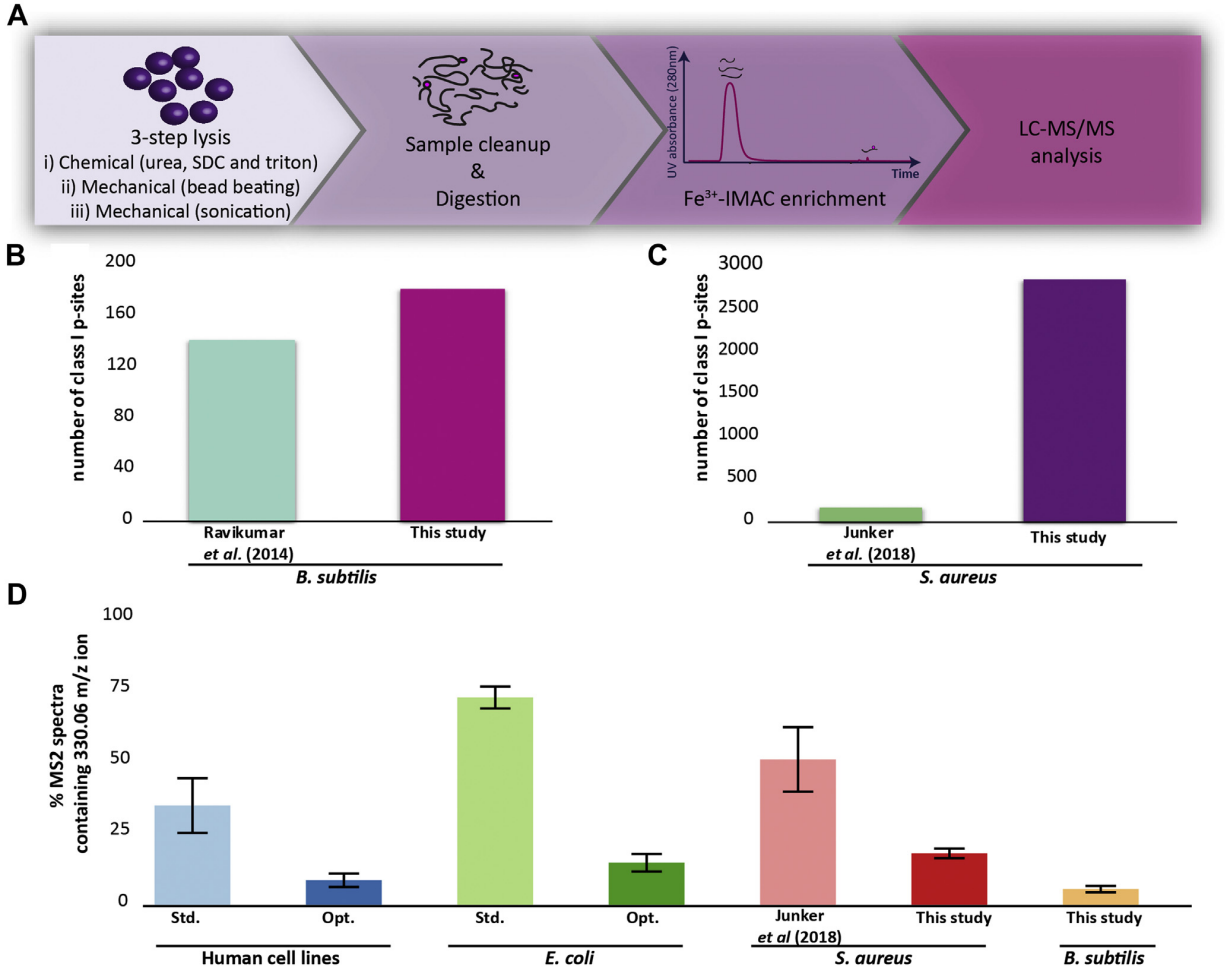
**Experimental workflow and literature comparison.***A*, cell lysis was optimized by introducing a three-step lysis using a mix of chemical and mechanical lysis. After sample cleanup and digestion, phosphopeptides were enriched using a Fe^3+^-IMAC column and analyzed *via* LC-MS/MS. *B*, the number of identified class I (Andromeda localization score > 0.75) phosphosites (176) for *B. subtilis* was compared with a recent study by Ravikumar *et al.* (29) and showed a 1.3× increase. *C*, in total, 2852 class I phosphosites were identified for *S. aureus* resulting in a 17-fold increase compared with Junker *et al.* (30). *D*, percentage of MS2 spectra containing the diagnostic ion 330.06 m/z for human cell lines, *E.coli*, *S. aureus*, and *B. subtilis*.

Reversible protein phosphorylation allows quick and effective signal transduction upon changing environmental conditions such as carbon accessibility or pH changes. This adaptation is especially important for bacterial colonization of different hosts but also after invasion and spread to different tissues. Recently, Potel *et al.* (11) optimized sample preparation for the phosphopeptide identification from Gram-negative bacteria. Here we present an extension of this protocol for application with Gram-positive bacteria, which contain a thick peptidoglycan layer and therefore require a more stringent lysis protocol. A three-step lysis protocol was developed, which combined chemical lysis using chaotropic agents and detergents with mechanical lysis by bead beating and subsequent sonication (see Experimental Procedures). After sample cleanup and protein digestion, phosphopeptides were enriched *via* Fe^3+^-IMAC and analyzed by LC-MS/MS (Fig. 1*A*). Optimization was initially tested on the Gram-positive model organism *B*. *subtilis* 168, before applying the optimized method to the lysis of the pathogenic bacteria *S*. *aureus* (transposon mutant NE98 of the strain *S. aureus USA300* JE3 containing a disruption in unrelated surface protein encoding gene *sdrE* (see Experimental Procedures)). The optimized lysis protocol for Gram-positive bacteria resulted in the identification of 283 phosphopeptides (pS, pT, pY, and pH) for *B. subtilis* (supplemental Fig. S1*A*, supplemental Table S2). When filtering on the Andromeda localization probability, 176 class I phosphosites on 146 proteins were identified in our study. This is a slight increase compared with the study of Ravikumar *et al.* (29) (Fig. 1*B*). For *S*. *aureus* the improvement was more striking. Here, 3800 phosphopeptides (pS, pT, pY, and pH) were identified in at least two out of three biological replicates, making it the largest phosphosite data set of *S. aureus* to date and representing a more than 21-fold increase compared with a recent study by Junker *et al.*, (30) which identified only 173 phosphopeptides (pS, pT, and pY) in *S. aureus* strain COL (supplemental Fig. S1*B* and supplemental Table S2). Also the identification of class I phosphosites (2852, Fig. 1*C*) was improved by more than 17-fold compared with previous work (30). To confirm that this improvement resulted from the reduction of DNA/RNA contamination, we looked for the diagnostic ion of 330.06 m/z in MS2 spectra. As previously demonstrated, the use of DNase and benzonase during sample preparation decreased the percentage of MS2 spectra containing the 330.06 m/z ion from around 35% to 8% for human samples and from 75% to 13% for *E. coli*, thereby enhancing phosphopeptide identification (11). For the Gram-positive bacteria, the amount of contamination was in a similar range; 3.5% of all MS2 spectra of *B. subtilis* and around 18% of all MS2 spectra of *S. aureus* contained the 330.06 m/z ion (Fig. 1*D*). In comparison, 50% of all MS2 spectra of the Junker *et al.* (30) study contained the 330.06 m/z ion (Fig. 1*D*), revealing a clear coenrichment of these contaminants. Therefore, the use of DNase and benzonase in our optimized sample preparation protocol improves phosphopeptide identification also for Gram-positive bacteria, by minimizing the binding of contaminants to the Fe^3+^-IMAC column.

### Phosphosite Distribution of S. aureus

**Fig. 2:**
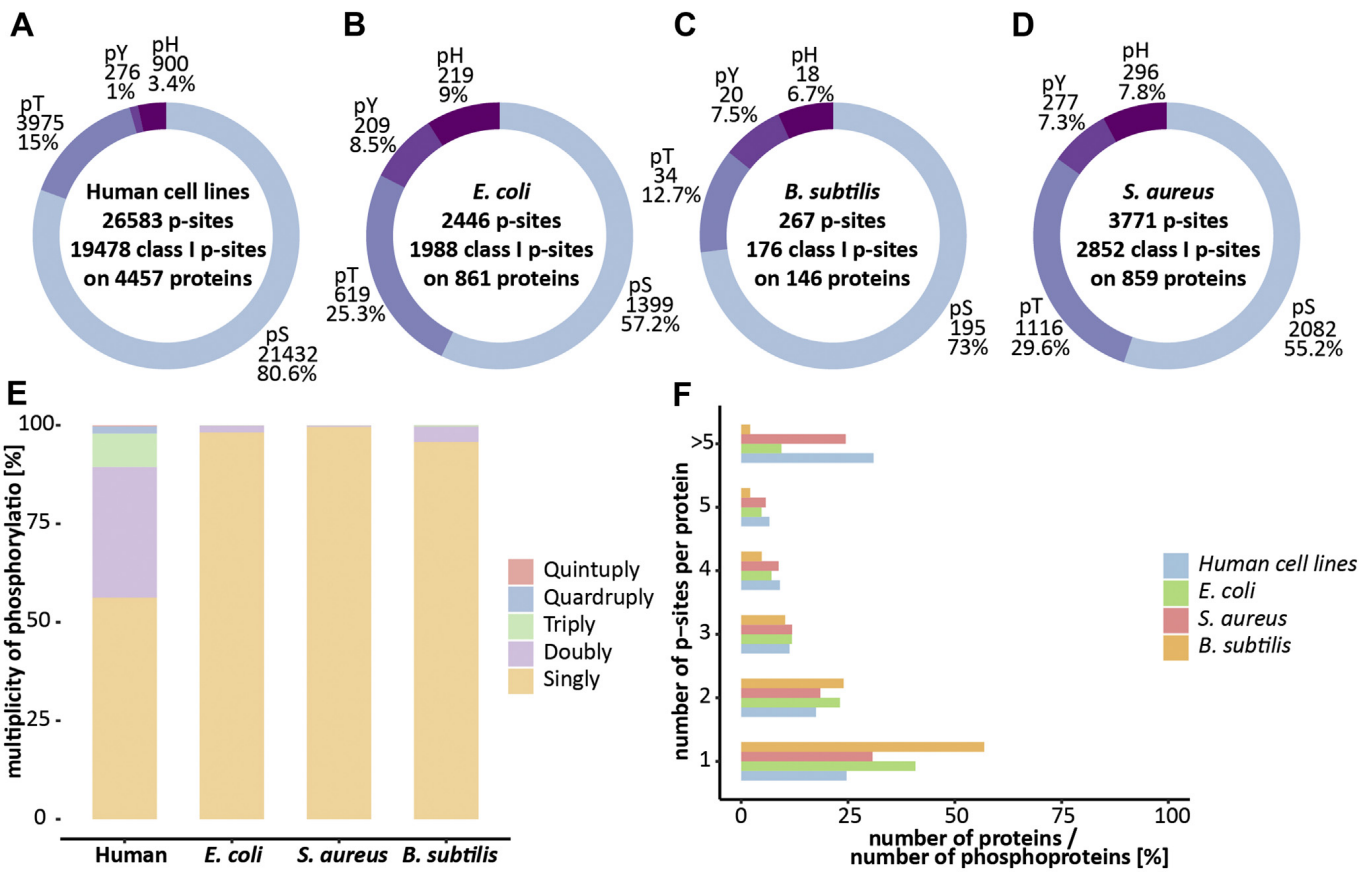
**Comparison of phosphorylation characteristic for human cell lines** (11)**, *E. coli*** (11)**, *B. subtilis*, and *S. aureus*.***A*–*D*, number of identified phosphosites, class I phosphosites (Andromeda localization probability >0.75) and phosphoproteins as well as the distribution of serine, threonine, tyrosine, and histidine phosphosites identified. *E*, percentage of singly, doubly, triply, quadruply, and quintuply identified phosphorylated peptides for all four organisms. *F*, percentage of phosphoproteins that have 1, 2, 3, 4, 5, or more phosphorylation sites for all four organisms.

The phosphosite distribution of eukaryotes and prokaryotes differs to a great extent. Whereas around 80 to 90% of all eukaryotic phosphorylation sites are localized on serine, phosphorylation on serine only accounts for 40 to 60% of all phosphorylation events in prokaryotes depending on the species (2, 31, 32). In addition to a clear shift toward threonine phosphorylation, prokaryotes also show a preference toward protein histidine phosphorylation compared with eukaryotes. We recently confirmed these preferences by identifying 80% of phosphorylation from human cell lines being localized on serine in contrast to only 57% of *E. coli* peptides (Fig. 2, *A* and *B*) (10). A similar preference was observed for *S. aureus*, where only 55% of all phosphorylation sites are localized on serine but almost 30% on threonine (55% pS, 29.6% pT, 7.3% pY, and 7.9% pH). Interestingly, *B. subtilis* showed a higher percentage of serine phosphorylation (73% pS, 12.7% pT, 7.5 pY, and 6.7% pH (Fig. 2, *C* and *D*), in agreement with previous phosphoproteomic studies on *B. subtilis* (29, 33). Not only the phosphosite distribution but also the multiplicity of phosphorylation differs between eukaryotes and prokaryotes (11). Here, we could confirm that the multiplicity of phosphopeptides for Gram-positive bacteria is similar to the Gram-negative bacterium *E. coli* and favors singly phosphorylated peptides. Indeed, we found that more than 90% of all identified prokaryotic phosphopeptides are singly phosphorylated (Fig. 2*E*), whereas eukaryotes showed a broad range of multiple phosphorylated peptides.

Interestingly, we could show that *S. aureus* also favored multiple phosphorylated proteins almost to the same extent as singly phosphorylated proteins, whereas *E. coli* and *B. subtilis* showed a high preference for singly phosphorylated proteins (Fig. 2*F*). Proteins with multiple phosphorylation sites are capable of generating different proteoforms, which are able to fulfill differential functions, which allows for a more complex and precise regulation of the pathogen (2). Previous work indicated that species with smaller genome sizes and lower number of bacterial transcription initiation factors (so-called sigma factors) display more multiple phosphorylated proteins, suggesting that regulation is mainly driven by PTMs in those bacteria (2). *S. aureus* indeed has considerable smaller genome and lower number of sigma factors compared with both *E. coli* and *B. subtilis.*

### Optimized Sample Preparation Allowed Identification of in Total 74 Potential Stk1 Targets

Even though it was long assumed that prokaryotes mainly exploit protein histidine phosphorylation comprised in TCSs, it is now obvious that Ser/Thr kinases play an important role in protein signaling as well (34). Depending on the strain, *S. aureus* comprises 16 to 18 TCSs but only one eSTKs (22, 23, 26). Therefore, we performed a phosphoproteome study to elucidate the targets of Stk1 as well as the cognate phosphatase Stp1. Hereto, the transposon mutants NE217 and NE1919 obtained from the Nebraska Transposon Mutant Library (NTML) (27) were used. NE217 and NE1919 have an inactivating disruption of the gene *pknB* (encoding Stk1) or the gene coding for the protein phosphatase 2C domain-containing protein Stp1. These mutants will be further referred to as Stk1 mutant and Stp1 mutant, respectively. NE98 was used as a control sample, allowing the analysis of phosphoproteome changes related to the activity of these two enzymes. Gene disruption and absence of those three proteins in the respective mutants were confirmed by PCR, full proteome as well as phosphoproteome analysis (supplemental Figs. S2 and S3).

**Fig. 3:**
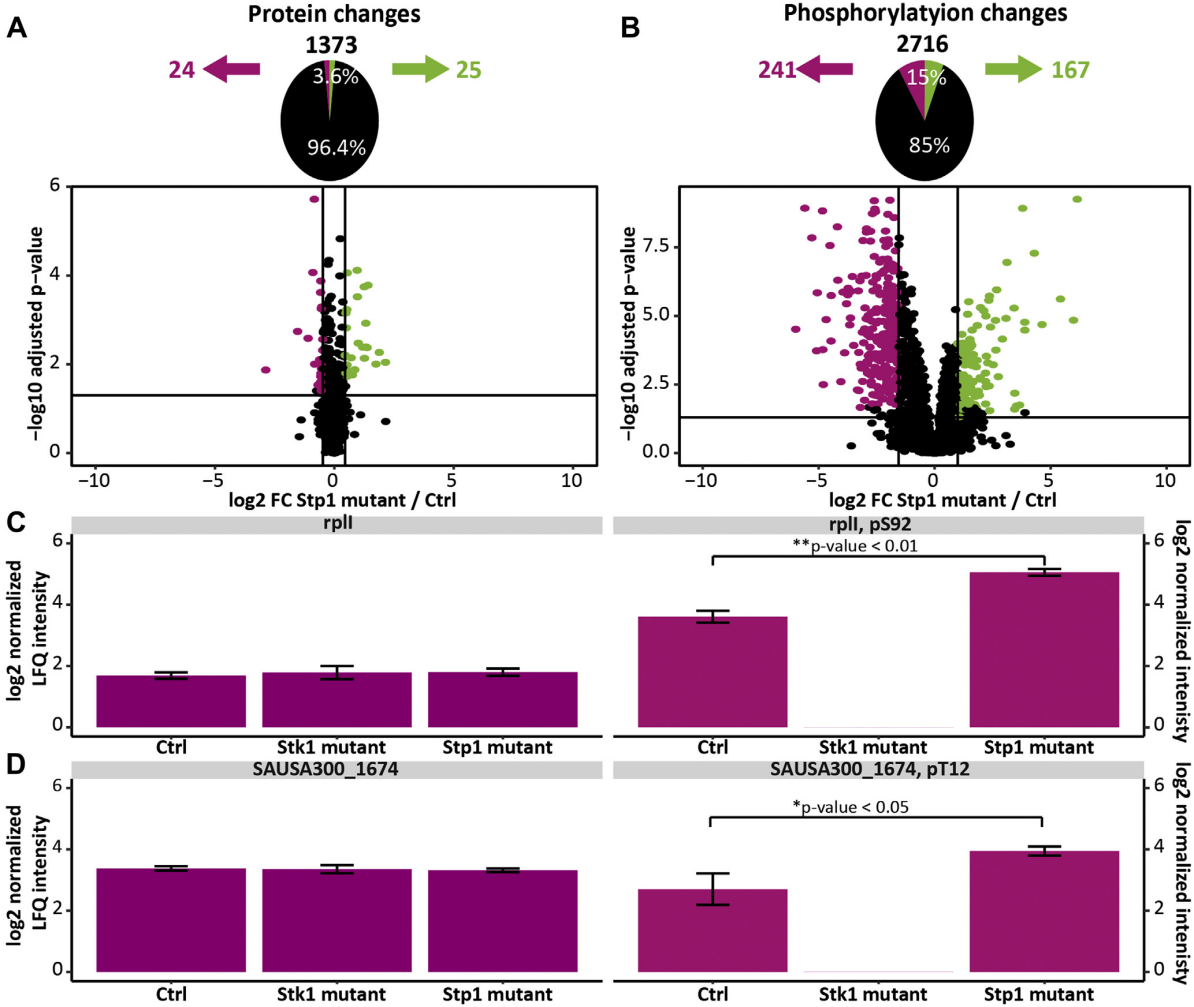
**Identified phosphorylation and protein changes in *S. aureus*.***A*, volcano plot comparing proteins between Stp1 mutant and Ctrl, identifying 51 proteins with significantly changing (Tukey HSD *p*-value cutoff of 0.05 and a fold change cutoff of $\bar{\text{x}}$ ± σ of the data). Twenty-five proteins were underrepresented (pink dots) and 26 proteins were overrepresented (*green dots*). *B*, volcano plot comparing the phosphorylation sites, identifying 408 changes between Stp1 mutant and Ctrl (Tukey HSD *p*-value cutoff of 0.05 and a fold change cutoff of $\bar{\text{x}}$ ± σ of the data). Two-hundred forty-one phosphosites were underrepresented (*pink dots*) and 167 phosphosites were overrepresented (*green dots*). *C*, overrepresentation of S92 phosphorylation in RplI in the Stp1 mutant compared with Ctrl (Tukey HSD *p*-value < 0.01) and absence of phosphorylation on the Stk1 mutant (*right panel*). No change in protein abundance was observed between the three mutants (*left panel*). *D*, overrepresentation of Thr12 phosphorylation on SAUSA300_1674 in the Stp1 mutant compared with Ctrl (Tukey HSD *p*-value <0.05) and absence of phosphorylation in the Stk1 mutant (*right panel*). On the proteome level, no significant changes were detected in the three mutants (*left panel*). For all bar charts, the center values represent the mean and error bars the standard deviation (s.d.) for n = 4 biological independent replicates.

To identify the changes upon *S. aureus* mutants, the three strains (n = 4 biological replicates) were cultured, lysed, and digested. Phosphopeptides were enriched using a Fe^3+^-IMAC column and analyzed *via* LC-MS/MS. Since Stk1 and Stp1 are being reported as a writer–eraser pair (35), potential targets of the kinase or phosphatase should show opposite behavior. Therefore, phosphosites overrepresented in the Stp1 mutant compared with the control and underrepresented or even absent in the Stk1 mutant and showing no significant change on the full proteome level are most likely to be substrates of these enzymes (supplemental Fig. S4). We identified and quantified 2716 phosphosites in both the Stp1 mutant and the control (see Experimental Procedures, Fig. 3, Supplemental Table S3). In total, 15% of these sites were significantly changing (Tukey HSD *p*-value cutoff of 0.05 and a fold change cutoff of $\;\bar{x}\pm \;\sigma$ of the data). In total, 241 of these phosphosites were significantly overrepresented and 167 phosphosites underrepresented in the Stp1 mutant compared with the control (Fig. 3*B*). On the full proteome level, 1373 proteins were quantified, of which 3.7% were significantly different between the two conditions (Fig. 3*A*, supplemental Table S4). Hence, most of the changes are occurring on the phosphoproteome level instead of the proteome level, which is in line with our expectations when Stk1 or Stp1 is absent. Phosphorylation on S92 of the 50S ribosomal protein L9 (rplI) showed a significant overrepresentation (Tukey HSD *p*-value < 0.01) in the Stp1 mutant compared with the control, whereas this phosphorylation could not be identified for the Stk1 mutant. On the full proteome level, this protein did not change significantly according to Tukey HSD (Fig. 3*C*). The same behavior was observed for the phosphosite pT12 of the putative serine protease HtrA (SAUSA300_1674, Fig. 3*D*). In total, 20 phosphosites were identified showing this characteristic (see Table 1 and supplemental Figs. S5 and S6). Moreover, 54 phosphosites were exclusively identified in Stp1 mutant, whereas the protein level was not affected significantly on the full proteome level or was not detected (Table 2). Thus, LFQ quantitative analysis of the three mutants (Stp1, Stk1, and Ctrl) allowed the identification 74 potential Stk1/Stp1-related phosphosites, which were distributed over the whole dynamic range of identified phosphopeptides as well as phosphoproteins (supplemental Fig. S7).

**Table 1:** Phosphosites overrepresented in Stp1 mutant/Ctrl but not identified in Stk1 without significant change on full proteome level

Gene	Uniprot ID	Protein name	Position within protein
ftnA	Q2FFK2	Bacterial nonheme ferritin	T98
map	A0A0H2XG88	Methionine aminopeptidase (MAP) (MetAP)	T153
rplI	Q2FKP1	50S ribosomal protein L9	S92
rplQ	Q2FER6	50S ribosomal protein L17	T33
ruvB	Q2FG86	Holliday junction ATP-dependent DNA helicase RuvB	S9
SAUSA300_0236	A0A0H2XGK7	PTS system, IIBC components	S475
SAUSA300_0905	A0A0H2XGC0	Putative adenylate cyclase	T3
SAUSA300_1230	A0A0H2XIS0	Uncharacterized protein	T5
SAUSA300_1240	A0A0H2XFT2	UPF0154 protein SAUSA300_1240	T80
SAUSA300_1674	A0A0H2XGV4	Putative serine protease HtrA	T12
SAUSA300_1856	A0A0H2XHR8	Uncharacterized protein	T89
SAUSA300_1909	A0A0H2XEY2	Uncharacterized protein	T143
secA1	Q2FIN8	Protein translocase subunit SecA 1	S702
secA1	Q2FIN8	Protein translocase subunit SecA 1	S565

**Table 2:** Phosphosites overrepresented in Stp1 mutant/Ctrl but not identified in Stk1 and no identification on full proteome level

Gene	Uniprot ID	Protein name	Position within protein
pknB	A0A0H2XGG5	Protein kinase	T166
pknB	A0A0H2XGG5	Protein kinase	T288
pknB	A0A0H2XGG5	Protein kinase	T314
SAUSA300_1574	Q2FGA9	UPF0297 protein SAUSA300_1574	T7
rplS HMPREF0776_2241	A0A0E1VS63	50S ribosomal protein L19 (Fragment)	T11

### Phosphoproteomic Analysis Reveals New Targets of STK1

We identified the sensor protein kinase HptS (part of the HptRS TCS) as a target of Stk1 (pT272, supplemental Table S3). Previous studies showed that Stk1 phosphorylates RRs of at least two TCSs: GraRS and VraRS (24, 25). With our identification of HptS as a specific target, we expand the repertoire of TCSs that are regulated by Stk1 to HptRS. But in contrast to GraRS and VraRS, in which the RR is phosphorylated, here Stk1 phosphorylates the sensor-like histidine kinase HtpS. HptRS belongs to the hexose phosphate transport system (HPT) that is partly responsible for the uptake of extracellular sugars that can vary between different host organisms (36, 37, 38, 39). Therefore, being able to switch quickly and efficiently between available carbon sources is critical for the survival and colonization of bacteria.

Transcription of HptRS is regulated by Catabolite control protein A (CcpA). CcpA has been reported to be phosphorylated in an Stk1-dependent manner on T18 and T33 in *S. aureus* strain N315^40^. Both phosphosites are located within the DNA-binding site of CcpA, and phosphorylation seems to interfere with DNA binding (40). Here, we identified eight serine and threonine phosphosites, including pT18, but none of those sites were significantly changing between the three mutants. Therefore, we hypothesize that Stk1/Stp1 is probably not the only regulator of phosphorylation of CcpA.

CcpA activation can also occur *via* a sugar dependent manner, which involves Histidine-containing protein (HPr) (40, 41, 42). In the presence of the primary carbon source glucose, HPr is phosphorylated by HPr kinase/phosphatase (HPrK/P) on S46. Only pS46-HPr is able to bind to CcpA, thereby enabling its binding to the catabolite response element (*cre*) on the chromosomal DNA, which can lead to either repression or activation of the respective gene transcription (38, 39, 42). pS46-HPr was identified in all three mutants without any significant change. However, phosphorylation of HPrK/P on S294 was identified exclusively in the Stp1 mutant, without changes in protein expression levels. Therefore, we suggest that phosphorylation of HPrK/P by Stk1 does not negatively influence its ability to phosphorylate HPr on S46. Even though Stk1 seems to phosphorylate important proteins within the CcpA regulatory machinery, we could not correlate those phosphorylation events to any changes in the abundance of proteins regulated by CcpA. Still, our data indicates extensive regulation of the *ccpA* regulon on multiple levels, which highlights its importance.

Finally, elongation factor tu (EF-Tu) was one of the proteins with phosphosites exclusively identified in the Stp1 mutant. EF-Tu was previously reported to be phosphorylated by Stk1 but without information on the phosphorylation site. Here, pT34 and pT257 were identified as two phosphosites on EF-Tu (supplemental Table S3). EF-Tu is one of the most abundant bacterial proteins that consists of three functional domains, domain I (amino acids 1–199) comprising the GTP/GDP binding domains, domain II (amino acids 200–299), and domain III (amino acids 300–394) that both regulate the activity of domain I (43, 44, 45). The main and most studied function of this protein is mediating protein translational elongation. Sajid *et al.* (46) showed that *Mycobacterium tuberculosis* EF-Tu is phosphorylated by an Stk1 homolog on 11 threonine residues. One of those, pT259, is homologous to pT257 identified in EF-Tu for *S. aureus*. However, Sajid *et al.* could not determine a negative effect of the phosphorylation on protein synthesis in *M. tuberculosis*. In our study, no growth defects on blood agar plates or in the liquid cultures using full media were observed for both Stk1 and Stp1 mutants, which is in line with previous studies (47). Given the essential role of EF-Tu in protein synthesis, it remains elusive how Stk1/Stp1 influence EF-Tu regulation.

Previous work has indicated that Stk1 and Stp1 mutants display defects in cell division as well as cell wall structure, affecting antibiotic susceptibility (48). With the identification of new Stk1/Stp1 substrates, future research can be more focused on how these target proteins and their phosphorylation sites are involved in these cellular processes.

### Extensive S/T Phosphorylation Indicates High S/T Kinase Activity in S. aureus Strain USA300

The identification of more than 3000 phosphosites localized on Ser and Thr (Fig. 1*D*) indicates that S/T kinase signaling and activity are much higher than previously anticipated. By deleting the only known eSTK, Stk1, we could still identify around 2700 phosphosites localized on Ser/Thr (see supplemental Fig. S8, supplemental Table S2). In addition, the quantification of only 74 potential Stk1 targets out of 2977 phosphosites localized on Ser or Thr raises the question of what the source of these other phosphosites is. The *S. aureus* strain USA300 has two other serine-protein kinases, HprK and RsbW. However, the number of currently reported targets for these kinases is low (49). This suggest that *S. aureus* USA300 must have other, currently unknown, Ser/Thr kinases.

In an effort to support this hypothesis, we tried to identify specific motifs and analyzed the flanking amino acids (± five amino acids) around pSer and pThr and compared this to well-studied human phosphosite data (HeLa phosphoproteome (11)). As expected, we could see a higher frequency for proline and serine adjacent to the phosphosites in human cell lines. This pattern was not observed in *S. aureus* and *E. coli* where acidic amino acids (aspartic and glutamic acid) as well as basic amino acids (lysine and arginine) were slightly more abundant adjacent to the phosphosite (see supplemental Fig. S9). Our analysis also directly showed that the identified phosphopeptides do not have an obvious motif, which was confirmed by the absence of enriched motifs using either iceLogo (50) or pLogo (51). Therefore, most of the identified phosphorylation events in *S. aureus* do not seem to occur within highly specific protein motifs. In addition, we were unable to obtain potential phosphorylation motifs using MEME (52). However, we cannot exclude that more specific motifs are buried in this large number of analyzed phosphosites.

**Fig. 4:**
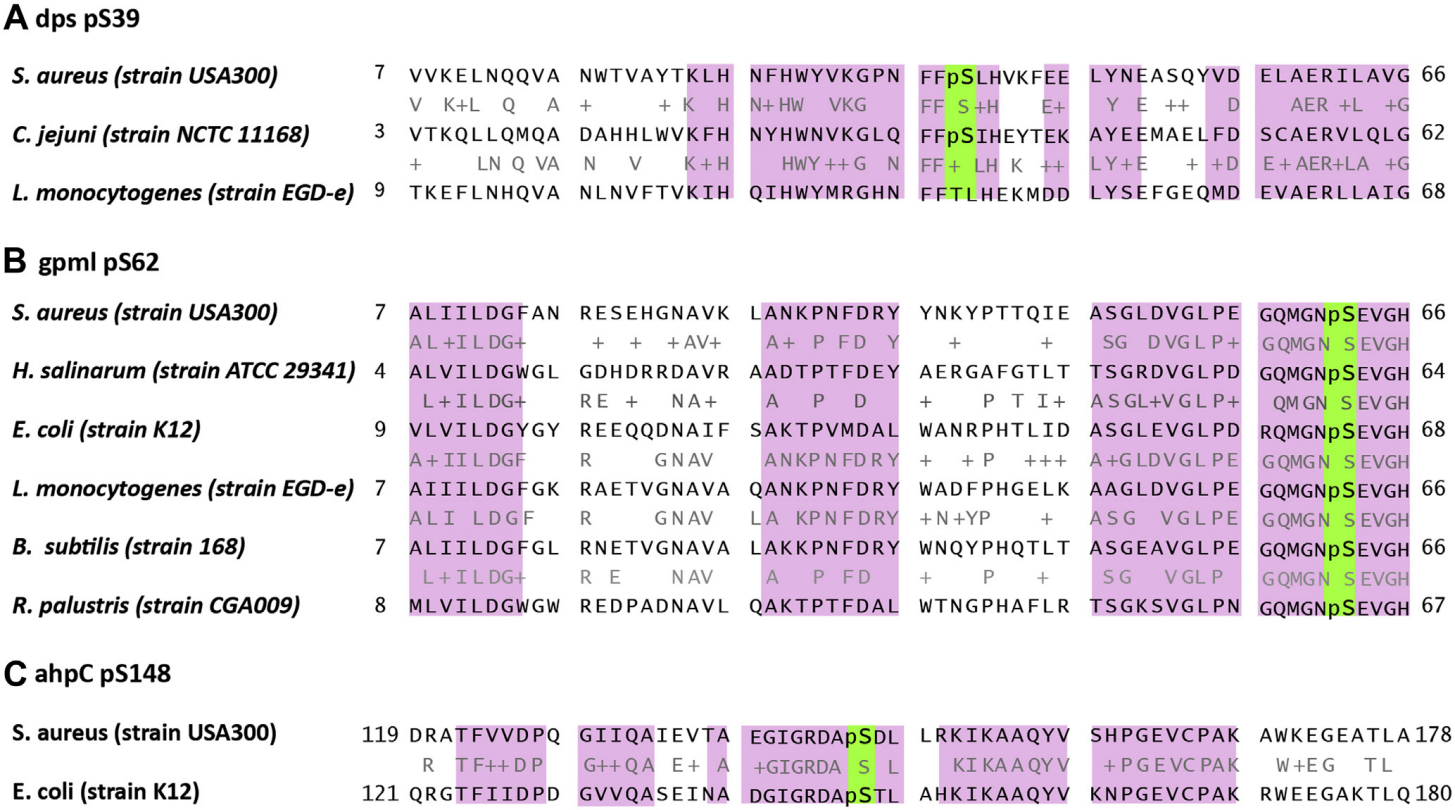
**Sequence alignments obtained with the NCBI Blastp (****https://blast.ncbi.nlm.nih.gov/Blast.cgi****) search for *A*, Dps, *B*, GpmI, and *C*, AhpC.** Conserved phosphosites are highlighted in *green* and regions of high conservation (more than five subsequent amino acids) are highlighted in *pink*. pS39 in Dps is conserved in *C. jejuni* and even though no phosphorylation is reported so far, *L. monocytogenes* contains S/T substitution at this position. pS62 in GpmI is conserved in *H. salinarum*, *E. coli*, *L. monocytogenes*, *B. subtilis*, and R. paulustris. pS148 in AhpC is conserved in *E. coli*. All three proteins show a high degree of conservation around the phosphosite.

**Fig. 5:**
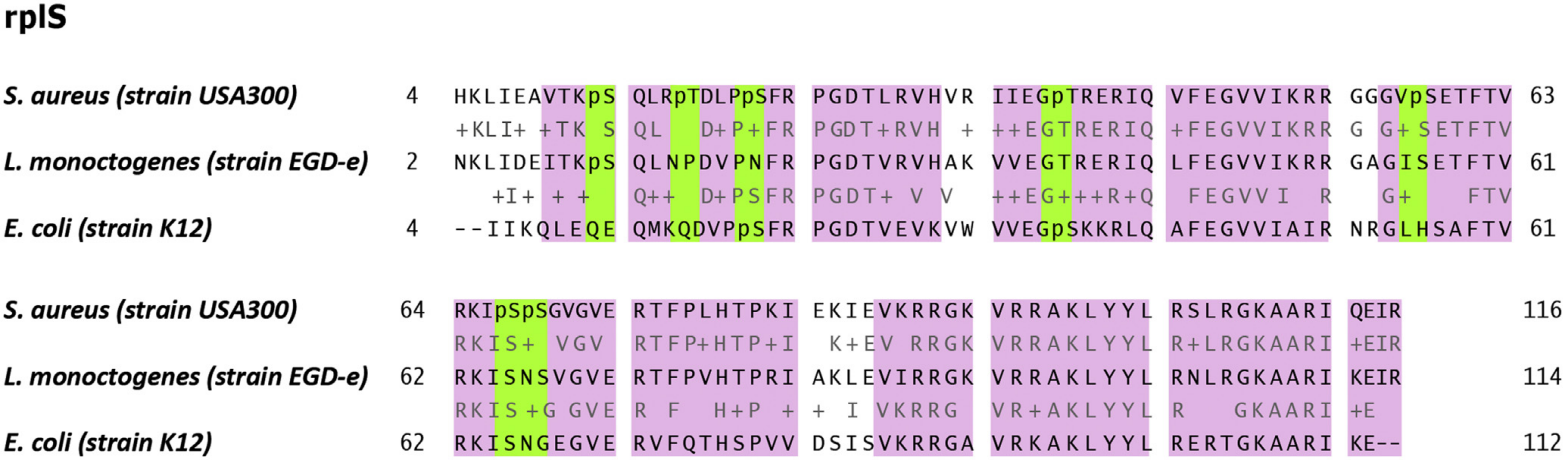
**Sequence alignment of RplS obtained with NCBI BlastP (****https://blast.ncbi.nlm.nih.gov/Blast.cgi****).** Ser/Thr phosphosites are highlighted in green and regions of high conservation (more than five subsequent amino acids) are highlighted in pink. In total we, identified seven phosphosites of which three (pS13, pS21, and pS38) were conserved according to dbPSP (32) and NCBI BlastP.

We also compared the identified pSer and pThr sites identified in this study with the entries in dbPSP (32). Here we could match 286 phosphoproteins based on their gene name. Proteins not having a gene name, but only a gene locus ID, were not included in the analysis. From the 286 genes, we identified 15 phosphosites that were conserved between different species. The alignments of those proteins show conservation hotspots (more than five subsequent amino acids, pink) especially localized around the conserved phosphosite (green) (Fig. 4, supplemental Fig. S10). One of those conserved phosphosites, pS62, on GpmI (Fig. 4*B*) is known to be a phosphoserine intermediate (Uniprot, UniRule: UR000100531); however, that was the only identified site reported as a metabolic phosphoserine intermediate. Even though we were only able to map 15 out of the more than 3000 identified phosphosites to already reported phosphorylation events, the observed conservation hotspots emphasized a high degree of conservation especially around the phosphosites. Further we could also identify conservation hotspots, for which we identified phosphosites in *S. aureus*, but no phosphorylation was detected in other species (Fig. 5). This rather low overlap in identified phosphosites but high conservation of certain regions is a reflection of the poor coverage of bacterial phosphoproteomes. This clearly highlights the importance of this study, in which improved sample preparation reveals an extreme underestimation of bacterial phosphorylation-mediated signaling. Despite the low overlap, our results still show that phosphorylation events are not just random even though specific motif were not identified. When looking for additional kinases by homology searches using NCBI protein Blast (https://blast.ncbi.nlm.nih.gov/Blast.cgi) or with motif searches using pfam (53), we did not obtain any results. Therefore, we recommend thorough characterization of the *S. aureus* proteome in order to identify novel kinases. Those kinases could play an important role in the virulence and versatility of this pathogen and hence identification of those kinases could help to understand and combat its pathogenicity.

## Data Availability

All raw data that support the findings of this study have been deposited in ProteomeXchange via the PRIDE partner repository with the dataset identifier PXD020226.

## Conflict of interest

The authors declare no competing financial interest.
