# Supplementary material for: In-Depth Characterization of the Staphylococcus aureus Phosphoproteome Reveals New Targets of Stk1
Source: Mol Cell Proteomics. 2021 Jan 11;20:100034. doi: 10.1074/mcp.RA120.002232 (PMC7950182; doi:10.1074/mcp.RA120.002232)
Supplement: Supplemental Figures S1–S10 [file mmc1.pptx]

## Slide 1
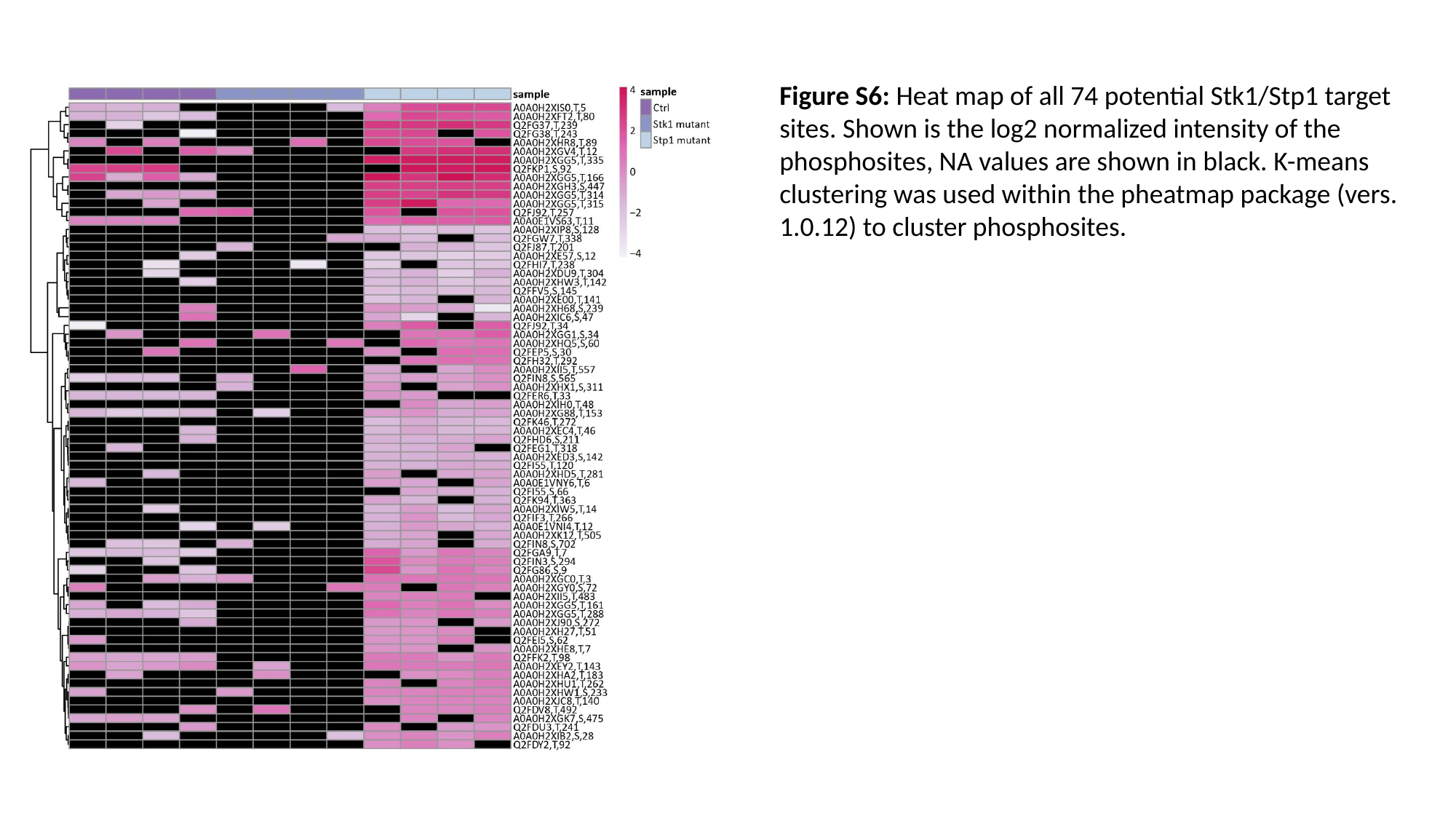

Figure S6: Heat map of all 74 potential Stk1/Stp1 target sites. Shown is the log2 normalized intensity of the phosphosites, NA values are shown in black. K-means clustering was used within the pheatmap package (vers. 1.0.12) to cluster phosphosites.
