# Supplementary material for: In-Depth Characterization of the Staphylococcus aureus Phosphoproteome Reveals New Targets of Stk1
Source: Mol Cell Proteomics. 2021 Jan 11;20:100034. doi: 10.1074/mcp.RA120.002232 (PMC7950182; doi:10.1074/mcp.RA120.002232)
Supplement: Supplemental Table S1 [file mmc2.docx]

Table S1: Fe3+-IMAC column enrichment gradient. Buffer A corresponds to the loading buffer (30 %ACN, 0.07 % TFA) and B to the elution buffer (0.3 % ammonia).

| **Time (min)** | **Flow rate (ml/min)** | **%A** | **%B** |
| --- | --- | --- | --- |
| 0 – 7.00 | 0.1 | 100 | 0 |
| 7.01 – 12.00 | 1 | 100 | 0 |
| 12.01 – 13.50 | 1 | 40 | 60 |
| 13.51 – 16.00 | 0.5 | 40 | 60 |
| 16.01 – 25.00 | 1 | 100 | 0 |
